# Immunohistochemical Detection of Tentonin-3/TMEM150C in Human Dorsal Root Ganglion, Cutaneous End-Organ Complexes, and Muscle Spindles

**DOI:** 10.3390/brainsci15040337

**Published:** 2025-03-24

**Authors:** Iván Suazo, Yolanda García-Mesa, José Martín-Cruces, Patricia Cuendias, Teresa Cobo, Olivia García-Suárez, José A. Vega

**Affiliations:** 1 Facultad de Ciencias de la Salud, Universidad Autónoma de Chile, Providencia 7500912, Chile; ivan.suazo@uautonoma.cl; 2 Grupo SINPOS, Departamento de Morfología y Biología Celular, Universidad de Oviedo, 33006 Oviedo, Spain; garciamyolanda@uniovi.es (Y.G.-M.); pepe3214@gmail.com (J.M.-C.); cuendiaspatricia@uniovi.es (P.C.); garciaolivia@uniovi.es (O.G.-S.); 3 Instituto de Investigación Sanitaria del Principado de Asturias, ISPA, 33011 Oviedo, Spain; 4 Departamento de Cirugía y Especialidades Médico-Quirúrgicas, Universidad de Oviedo, 33006 Oviedo, Spain; 5 Instituto Asturiano de Odontología (IAO), 33006 Oviedo, Spain

**Keywords:** tentonin-3/TMEM150C, mechano-gated ion channels, mechanoreceptors, dorsal root ganglia, cutaneous end-organ complexes, muscle spindles, human

## Abstract

Background/Objectives: Tentonin-3/TMEM150C is a pore-forming protein of a mechanically activated channel recently identified that typically displays rapid activation followed by slow inactivation. It has been detected in murine dorsal root ganglia, nodose ganglion baroreceptors, and muscle spindles. Nevertheless, primary sensory neurons expressing tentonin-3/TMEM150C fall into the categories of nociceptors, mechanoreceptors, and proprioceptors. Methods: We used immunohistochemistry and image analysis (examining the size of the neuronal bodies in the dorsal root ganglia) to investigate the distribution of tentonin-3/TMEM150C in human cervical dorsal root ganglia, sensory nerve formations in the glabrous skin, especially cutaneous end-organ complexes or sensory corpuscles, and muscle spindles. Results: In dorsal root ganglia, 41% of neurons were tentonin-3/TMEM150C-positive, with a distribution of small (12.0%), intermediate (18.1%), and large (10.9%). In the glabrous skin, tentonin-3/TMEM150C was observed in the axon of Meissner, Pacinian, and Ruffini corpuscles as well as in the axon of the Merkel cell–axon complexes. Furthermore, tentonin-3/TMEM150C-positive axons were observed in muscle spindles. No free nerve endings displaying immunoreactivity were found. Conclusions: This is the first report on the distribution of tentonin-3/TMEM150C immunoreactivity in the human peripheral somatosensory system, and although it is a brief preliminary study, it opens new perspectives for the study of this new mechano-gated ion channel.

## 1. Introduction

Mechanical forces, like tension, pressure, vibration, etc., produce movements in the skin that activate specialized structures collectively known as cutaneous end-organ complexes (CEOCs) or sensory corpuscles [1,2,3,4]. The axons innervating CEOCs are the peripheral processes of low-threshold mechanoreceptors (LTMRs) whose bodies are in the dorsal root ganglia (DRG) [5]. Inside of the CEOCs, mechanotransduction occurs; that is, the conversion of the mechanical stimuli into action potentials. Similarly, mechanotransduction also occurs in the proprioceptor endings of muscle spindles, which are stretch detectors [6]. In humans, sensory terminals of proprioceptors form irregular coils with branches and varicose swellings [7].

Among proteins that can be modified by forces are transmembrane proteins that form an integral part of ion channels [8,9] that act together with cytoskeletal proteins and components of the extracellular matrix [10,11]. All these molecules are mechanosensors and are organized into complexes responsible for both the mechanosensing and mechanotransduction pathways. At present, the only ion channels that fully satisfy the conditions as mechanotransducers are the PIEZO channels, PIEZO1 and PIEZO2, which are mechanically activated channels with rapid inactivation [12,13,14]. Recently a new mechanically activated ion channel named tentonin-3/TMEM150C (TTN3) has been identified [15,16]. TTN3 is a pore-forming subunit of a mechanically activated channel, present throughout vertebrates, that typically displays activated currents in response to mechanical step stimuli: a rapid activation followed by a slow inactivation [17].

In murine DRG, TTN3-positive neurons fall into the categories of mechanoreceptors, proprioceptors, and nociceptors based on the co-localization of TTN3 with specific markers for each category [15]. On the other hand, in the murine nodose ganglion, functional studies have demonstrated that TTN3-positive neurons are rapidly adapting (RA)-type, intermediary adapting (IA)-type, and slowly adapting (SA)-type neurons [18]. Therefore, the TTN3-positive neurons of the DRGs potentially project onto the skin to form CEOCs and free nerve endings, as well as onto the muscle spindles of the skeletal muscles. TTN3 was detected in muscle spindles [15] and the endings of the baroreceptors (a subtype of mechanoreceptor) [18,19] but never in CEOCs. Consistent with the patterns of TTN3 expression, the genetic deletion of the *Ttn3* in the DRG and the nodose ganglion (where the bodies of the aortic sinus baroreceptors are localized) [18,19] ablates the mechanically activated SA-type currents. Also, mechanically activated TTN3 currents are inhibited by known blockers of mechanosensitive ion channels [15,18]. However, and despite all this evidence, recently published literature shows conflicting data suggesting that TTN3 does not contribute to mechanosensitivity [20,21,22].

Since DRG TTN3-expressing neurons show distinct levels of adaptation to mechanical stimuli, and immunohistochemical studies suggest that they are mechanoreceptors, proprioceptors, and nociceptors, we hypothesized that TTN3 could also be present in the peripheral projections of all these neurons. Here, we used immunohistochemistry to analyze the localization of TTN3 in the human cervical DRG, digital cutaneous CEOCs, and muscle spindles. This study aims to contribute to the knowledge of human mechanosensitivity, especially that which is related to the sense of touch.

## 2. Materials and Methods

### 2.1. Material

The material used in this study was obtained from the SINPOS research group histological archive (Department of Morphology and Cell Biology of the University of Oviedo; National Registry of Biobanks, Collections Section, Ref. C-0001627) and was obtained in compliance with Spanish legislation (RD 1301/2006; Law 14/2007; DR 1716/2011; Order ECC 1414/2013). Tissues were from healthy subjects that suffered finger amputation or died in traffic accident and whose organs were used for transplantation. The pieces were fixed in 10% buffered formalin, washed in tap water for 12 h, rinsed in distilled water for 1 h, and routinely embedded in paraffin. The material included samples of cervical spinal ganglia (*n* = 8, from 3 males, with ages ranging between 39 and 61 years) and glabrous digital skin (*n* = 14, with ages ranging between 21 and 68 years) and samples of skeletal muscle (*n* = 3; *biceps brachii* muscle) used to immunolabel muscle spindles that were used as positive controls.

### 2.2. Immunohistochemistry

Rehydrated sections were heated in Envision FLEX target retrieval solution high pH (Dako, Glostrup, Denmark) at 65 °C for 20 min and then for 20 min at room temperature in the same solution, followed by washing in TBS (tris buffered solution, pH 7.4) containing 1% Triton X100, and then the endogenous peroxidase activity was inhibited with a buffered solution of 3% H_2_O_2_. The sections were then washed in the same buffer as above and non-specific binding was prevented by incubating with 10% fetal calf serum albumin (F2442, Sigma-Aldrich, Saint Louis, MO, USA). Thereafter, the sections were incubated overnight at 4 °C in a humid chamber, with two different rabbit polyclonal antibodies: anti-tentonin-3/TMEM150C (extracellular) antibody peptide (C)EDDKILPLNSAARKSGVK, corresponding to amino acid residues 33–50 of rat TMEM150C (APC-088, Alomone Labs., Jerusalem, Israel), and anti-tentonin 3/Tmem150C, a linear peptide corresponding to 38 amino acids from the C-terminal region of murine transmembrane protein 150C (ABN2266, Sigma-Adrich). Both antibodies were used diluted at a level of 1:100 and have been reacted with rat and mouse tissues and have not been assessed on human tissues (according to the manufacturer’s notice). However, since the TTN3 orthologs (including mouse, human, zebrafish, cat, and chick) demonstrated typical rapid activation followed by rapid and slow inactivation MA currents in response to mechanical step stimuli, a similar amino acid sequence between the TTN3 of vertebrates is hypothesized. After incubation with the primary antibodies, the sections were washed in TBS and incubated at room temperature with Dako EnVision System labeled polymer-HR anti-rabbit IgG (DakoCytomation, Glostrup, Denmark). Finally, sections were rinsed in distilled water and the immunoreaction visualized using 3-3′-diaminobenzidine as a chromogen.

For control purposes, representative sections were processed as above using non-immune rabbit serum instead of the primary antibodies or omitting the primary antibodies in the incubation. Under these conditions no specific immunostaining was detected.

### 2.3. Quantitative Image Analysis

A quantitative image analysis was conducted in DRG sections reacted for TTN3 immunodetection using an automatic image analysis system (Quantimet 550, Leica, QWIN Program). The percentage and size (mean diameter in μm) of the immunoreactive neurons were evaluated. Measurements were made on three sections per specimen, 200 μm apart to avoid measuring the same neuron twice, evaluating five randomly selected fields per section (2.5 mm^2^ × 24 sections). For evaluation of the neuron size, only the cell profiles with apparent nuclei were considered. The neurons were divided into 3 categories according to their diameter: ≤20 µm (small neurons, regarded to be nociceptors), 21–50 µm (intermediate neurons, regarded to be mechanoreceptors), and >50 µm (large neurons, considered be proprioceptors). The results are expressed as mean ± standard deviation and refer to the variation among samples.

## 3. Results

The study to detect TTN3 immunoreactivity was conducted on formations related to the peripheral somatosensory system, including DRG, glabrous skin, and muscle spindles. Considering that the antibodies used are not specific against human TTN3, it would be more appropriate to write “immunoreactivity-like” instead of “immunoreactivity” throughout the manuscript, but we have omitted “like” in favor of fluent reading.

### 3.1. TTN3 Immunoreactivity in Human DRG

**Figure 1:**
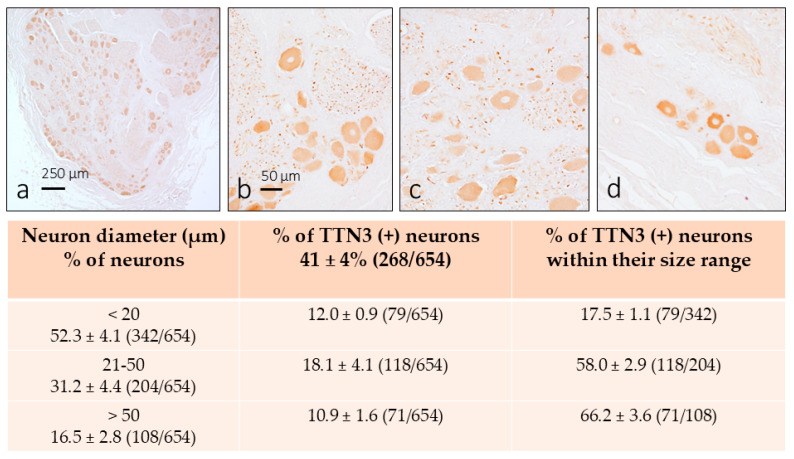
Immunohistochemical detection of TTN3 in the human dorsal root ganglion C5 (**a**) within the three pre-established size ranges. A subpopulation of sensory neurons displayed TTN3 immunoreactivity as well as some intraganglionic axons. The table shows the results of the quantitative analysis. The scale bar in (**b**) is identical for (**c**,**d**).

In the first step of the study, we investigated the occurrence of TTN3 in the cervical human DRG and in which category the TNN3-positive neurons fell within the three pre-established body sizes. TTN3 immunoreactivity was observed in all analyzed sections, covering the entire range of the neuronal diameters. In addition to the neuronal soma, positive immunoreactivity was detected in some intraganglionic axon profiles (Figure 1a–c). Within the neuronal cytoplasm, the pattern of immunoreactivity was homogeneous, although the intensity of immunostaining varied among neurons independently of the size. No immunoreactivity was observed in the satellite glial cells.

The percentage of TTN3-positive neurons was about 41 ± 4% (265/654 evaluations) of the total counted neurons and were distributed across the pre-established size categories as follows: 12.0% small neurons (79/654), 18.1% intermediate neurons (118/654), and 10.9% (71/654) large neurons (see the table in Figure 1). The percentage of neurons TNN3-positive with respect to the total number of neurons in each pre-established size range were 17.5% of small neurons, 58% of intermediate neurons, and 66.2% of large neurons (see the table in Figure 1). Based on neuron size alone, the functional properties of the TTN3-positive DRG neurons can be attributed approximatively to mechanoreceptors and proprioceptors and, to a lesser extent, to nociceptors. No immunoreactivity for TTN3 was observed in control sections.

### 3.2. TTN3 Immunoreactivity in Human Digital CEOCs

Although our research on the skin focuses on CEOCs and Merkel cell–axon complexes, dermal nerves have also been studied. TTN3 immunoreactivity was detected in a subpopulation of nerve fibers, marking axons that were of a larger caliber. Neither in the dermis nor the epidermis were free-nerve endings found with a positive reaction for TTN3.

**Figure 2:**
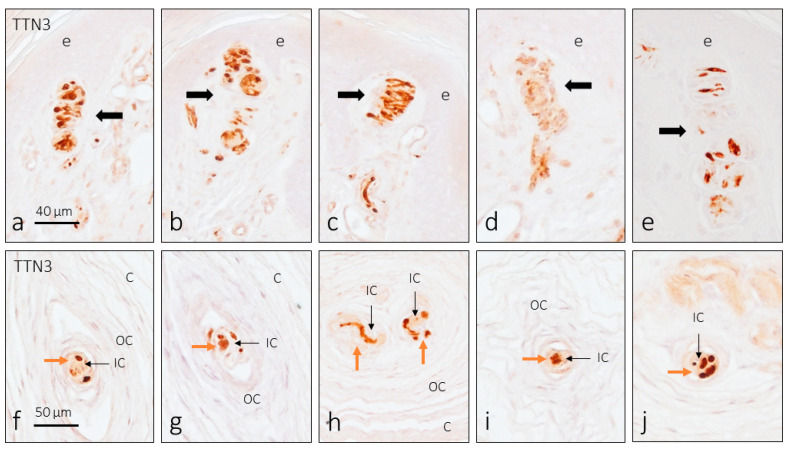
Immunohistochemical detection of TTN3 in the human cutaneous Meissner (**a**–**e**, arrows) and Pacinian (**f**–**j**) corpuscles. In images (**f**–**j**), the brown arrows indicate TTN3 immunoreactive axons whereas the black arrows indicate the inner core of the corpuscles. e: epidermis; C: capsule; IC: inner core; OC: outer core. The scale bar in (**a**) is identical from (**a**–**e**); the scale bar in (**f**) is identical from (**f**–**j**).

**Figure 3:**
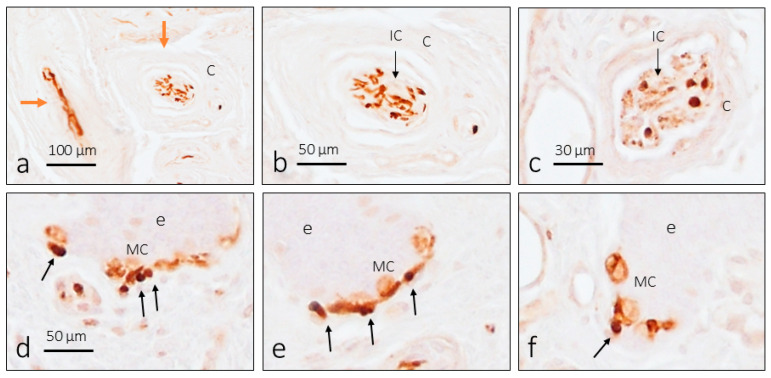
Immunohistochemical detection of TTN3 in the human cutaneous Ruffini’s corpuscles (**a**–**c**, arrows) and Merkel cell–axon complexes (**d**–**f**) corpuscles. The brown arrows indicate one Pacinian corpuscle (left) and one Ruffini’s corpuscle (right). The black arrows in (**d**–**f**) indicate TTN3 immunoreactive axon profiles. e: epidermis; C: capsule; IC: inner core; MC: Merkel cells. The scale bar in (**d**) is identical for (**d**–**f**).

The distribution of TTN3 immunoreactivity in digital skin affects all CEOC morphotypes (Figure 2 and Figure 3). In Meissner corpuscles, immunolabeling was clearly detected in the axon (Figure 2a–e) and only in a low proportion of corpuscles (less than 2%) was there weak immunoreactivity in the terminal glial cells, i.e., the lamellar cells (Figure 2d). The same pattern of axon immunoreactivity was observed in Pacinian corpuscles (Figure 2f–j). Some Pacinian corpuscles showed more than one axon profile, although it is not certain whether they are several afferents or the arborization of a single axon. In these CEOCs, no TTN3 immunoreactivity was observed in the terminal glial cells that form the inner core. Finally, the immunolocalization of TTN3 in Ruffini’s corpuscles was always restricted to the axon (Figure 3a–c).

### 3.3. TTN3 Immunoreactivity in Human Digital Merkel Cell–Axon Complexes

In addition to the CEOCs, TTN3 immunoreactivity was detected in nerve profiles at the base of the epidermal crests associated with cells identified as Merkel cells (based on their location and morphology). Therefore, the axons of the sensory Merkel cell–axon complexes display TNN3 immunoreactivity (Figure 3d–f).

### 3.4. TTN3 Immunoreactivity in Muscle Spindles

**Figure 4:**
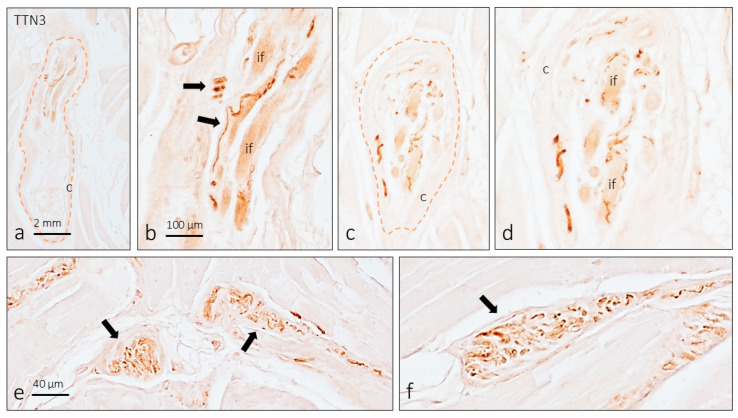
Immunohistochemical detection of TTN3 in the human muscle spindles, bounded by brown dotted lines (**a**–**d**; (**b**) and (**d**) are details of (**a**) and (**c**), respectively). Arrows in (**b**) indicate TTN3-positive nerve fibers. Perimysium nerve formations displaying TTN3 immunoreactivity were found ((**e**,**f**), arrows). The scale bar in (**a**) and (**b**) are identical for (**c**) and (**d**), respectively; the scale bar in (**e**) is identical for (**f**).

The only sensory nerve formations in which immunoreactivity for TTN3 has been described are mouse muscle spindles. In longitudinal sections of *biceps brachii*, muscle spindles (five in total) were found, in which TTN3 immunoreactivity was observed in axon profiles in relation to intrafusal muscle fibers (Figure 4a–d). In addition, positive TTN3-positive nerve profiles and non-typical sensory nerve formations were also observed in the septa of perimysium (Figure 4e,f).

No age- or sex-dependent changes were found in the distribution of TTN3 immunoreactivity in any of the tissues studied. Likewise, no differences in the pattern or intensity of immunostaining were observed between the two antibodies used.

## 4. Discussion

The present study was designed to establish the distribution of TTN3, a protein forming a part of one mechano-gated ion channel, in the human peripheral somatosensory system. The study included DRGs, sensory nerve formations, especially CEOCs, in the skin, and neuromuscular spindles of striated muscles. To map TTN3 in these tissues, we used immunohistochemistry. The results obtained, although clear and evident, should be taken with caution since non-specific antibodies for human TTN3 have been used.

Our findings in human DRG matched those found by Hong et al. [15] in the murine DRG. Because TTN3 is a part of a mechanosensitive ion channel, one would think that it is expressed exclusively in mechanoreceptors. However, it was detected in neurons of the entire size range and, therefore, in nociceptors, mechanoreceptors, and proprioceptors. Comparable results were obtained by Hong et al. [15] who co-localized TTN3 with the large unit of the neurofilament (mechanoreceptor marker), parvalbumin (proprioceptor marker), and TRPV1 (transient receptor potential vanilloid 1; nociceptor marker). Here, we have not used double immunolabeling to identify the subtypes of neurons in the DRGs, but we have done so according to the size of the neuronal soma. Physiological studies on the mammalian DRG have determined that intermediate- and large-sized neurons are cutaneous and muscular mechanoreceptors, while small neurons are nociceptive [5,23,24,25]. Regarding the speed of adaptation, no data exist involving DRG but TTN3-positive neurons of the mouse nodose ganglion were rapidly adapting (RA)-type, intermediary adapting (IA)-type, and SA-type neurons [18].

The only available information about the occurrence of TTN3 in the peripheral process of the pseudo-unipolar axon of the DRGs is its presence in the axons that supply the murine muscle spindles [15]. Our results in humans are in complete agreement with those in mice but, based on present findings alone, it is not possible to know whether it is afferent fibers Ia or II [6].

There is no information on the presence of TTN3 in CEOCs or other endings of mechanoreceptors in the glabrous skin. In our hands, the axons of Meissner, Pacinian, and Ruffini corpuscles, as well as the axons of the Merkel cell–axon complexes, displayed TTN3 immunoreactivity. All of these cutaneous sensory nerve formations are associated with Aβ-LTMRs. LTMRs can be further classified based on electrophysiological and histological characteristics, neurochemical properties, and molecular markers, as well as according to the morphologies of their peripheral endings [26,27]. LTMRs fall into two categories: rapidly adapting (RA) LTMRs and slowly adapting (SA) as well as a less typified group of “intermediate adapting” LTMRs. Both RA and SA LTMRs, in turn, have type I and type II RA type I and II forms at periphery Meissner and Pacinian corpuscles, respectively, while SA type I forms Merkel cell–neurite complexes and SA type II forms dermal Ruffini’s corpuscles [1,2,28]. In addition to LTMRs, there are nociceptors encoding forces in the noxious range.

Our results seem to contradict in part the studies that identify TNN3 as a part of a slowly adapting mechano-gated ion channel. The distribution in DRG mechanoreceptors, Merkel cell–axon complexes, and Ruffini corpuscles lends support to this interpretation. However, the detection of TTN3 in the axon of Meissner and Pacinian’s corpuscles, which are rapidly adapting, suggests that it is also involved in rapidly adapting processes. Therefore, our findings suggest that TTN3 may be involved in the slow and fast adaptation processes of adaptation to mechanical stimuli. But further studies are necessary to establish whether TTN3 is a mechanosensor or mechanotransducer protein *per se* or whether its activity depends on the presence of other ion channels such as PIEZO1 or PIEZO2. In fact, TTN3 and PIEZO2 are widely co-located in sensory neurons [18], and TTN3 is considered a modulator of PIEZO1, not an independent mechanically activated channel, because TTN3 is not activated by mechanical stimuli in the absence of PIEZO1 [20,29].

This study aimed to report that TTN3 immunoreactivity in humans is present not only in DRGs and neuromuscular spindles but also in the peripheral endings of cutaneous mechanoreceptors. The present data should be the beginning of future studies aimed at definitively characterizing TTN3-positive sensory neurons by double immunolabeling. Nevertheless, the markers must be carefully selected since, for example, neurofilament immunoreactivity in adult human DRG, in contrast to rodents, is not restricted to larger neurons but is present in virtually all DRG neurons [30,31]. Furthermore, studies are in progress in our laboratory to verify the co-localization of TTN3 with PIEZO1 or PIEZO2 in DRGs and CEOCs.

## 5. Conclusions

The immunohistochemical study on the distribution of TTN3 in humans corroborates previously described data regarding its association with slowly adapting mechanoreceptors (Merkel cell–neurite complexes and Ruffini corpuscles) but also, for the first time, demonstrates its presence in rapidly adapting CEOCs (Meissner and Pacinian corpuscles). These data suggest that TTN3 could be involved in both types of adaptation to mechanical stimuli; moreover, its co-localization with the mechanoproteins PIEZO1 and PIEZO2 appears to indicate a modulatory role for them rather than an independent function as previously described. Additional studies are necessary to determine whether TTN3 is a mechanosensor by itself or if its activity depends on direct interaction with other ion channels.

## Data Availability

The data that support the findings of this study are available from the corresponding author upon reasonable request.

## Abbreviations

The following abbreviations are used in this manuscript:

TTN3	Tentonin-3/TMEM150C.
CEOCs	Cutaneous end-organ complexes.
DRG	Dorsal root ganglia.
LTMRs	Low-threshold mechanoreceptors.
